# New Forms of Neuroactive Phospholipids for DHA Enrichment in Brain

**DOI:** 10.3390/md22030116

**Published:** 2024-02-29

**Authors:** Romina Gomes, Inês Mendes, Maria Paula Duarte, Narcisa M. Bandarra, Ana Gomes-Bispo

**Affiliations:** 1 Division of Aquaculture, Upgrading and Bioprospection (DivAV), Portuguese Institute for the Sea and Atmosphere (IPMA, I.P.), Avenida Alfredo Magalhães Ramalho, 6, 1495-165 Algés, Portugalines.s.mendes@estudantes.ips.pt (I.M.);; 2 MEtRICs/Departamento de Química, NOVA School of Science and Technology|FCTNOVA, Universidade Nova de Lisboa, Campus de Caparica, 2829-516 Caparica, Portugal; mpcd@fct.unl.pt; 3 Barreiro School of Technology, Polytechnic Institute of Setubal, Rua Américo da Silva Marinho, 2839-001 Lavradio, Portugal; 4 Interdisciplinary Centre of Marine and Environmental Research (CIIMAR), University of Porto, Rua dos Bragas 289, 4050-123 Porto, Portugal

**Keywords:** Atlantic mackerel (*Scomber scombrus*), phospholipids, lysophosphatidylcholine, docosahexaenoic acid (DHA), Alzheimer’s disease (AD)

## Abstract

Low levels of docosahexaenoic acid (DHA) in the brain have been related to neurological disorders, like Alzheimer’s disease (AD). After ingestion, dietary DHA must cross the blood–brain barrier, where it is absorbed as lysophosphatidylcholine (LPC), due to its role as a preferential DHA carrier in the brain. This work aimed at the production of LPC-DHA extracts to be used in supplementation/food fortification intended neural enrichment in DHA. As it is rich in DHA, especially its phospholipids (PL), Atlantic mackerel (*Scomber scombrus*, caught in Spring/2022) was used as a raw material. The polar lipids fraction was separated and hydrolysed with *Rhizomucor miehei* lipase, to enzymatically convert phosphatidylcholine (PC) into LPC. The fish (muscle and by-products) lipids fraction was used for total lipids (TL) content, lipid classes (LC) and fatty acid (FA) profile evaluation, whilst polar lipids extracts were studied for LC production and FA analysis. Muscle TL ranged between 1.45 and 4.64 g/100 g (WW), while by-products accounted for 7.56-8.96 g/100 g, with the highest contents being found in March. However, PL were more abundant in muscle (22.46–32.20% of TL). For polar lipids extracts, PL represented 50.79% of TL, among which PC corresponded to 57.76% and phosphatidylethanolamine to 42.24%. After hydrolysis, nearly half of this PC was converted into LPC. When compared to the initial PC, DHA relative content (33.6% of total FA) was significantly higher after hydrolysis: 55.6% in PC and 73.6% in LPC. Such extract, obtained from this undervalued species, may represent a promising strategy to increase DHA uptake into brain cells while allowing this species to upgrade.

## 1. Introduction

Worldwide, the prevalence of dementia is increasing, representing a heavy burden for national health systems. According to Wong et al. [1], it is estimated that in 2020, the burden of total healthcare costs for the treatment of Alzheimer’s disease (AD) corresponded to USD 305 billion. This value, however, may rise up to USD 1 trillion as the population ages.

The pathologic hallmarks of AD include the accumulation of extracellular amyloid-β plaques (Aβ), intracellular neurofibrillary (tau) tangles, and reduced glucose uptake in the brain [2,3]. In particular, the peptides Aβ40 and Aβ42 are involved in this pathological process, and for that reason, they are considered AD biomarkers [3,4].

Altered lipid metabolism in the brain is another characteristic of AD [5]. It is known that lipids correspond to about 50–60% of the human brain’s dry weight, of which nearly 30% corresponds to polyunsaturated fatty acids (PUFA) [2,5]. Docosahexaenoic acid (22:6 n-3, DHA) corresponds to half of the brain PUFA and is recognised to have an important role in brain health. Indeed, a deficiency of DHA has been linked to cognitive decline during ageing, as well as to the occurrence of several neurological disorders including AD [6,7]. Low DHA levels were found to occur particularly in regions affected by AD, such as the hippocampus [8].

Since neurons lack the ability to synthesize DHA, it must be acquired through the diet and transported from the plasma across the blood–brain barrier (BBB) into the brain [2]. In fact, there is increasing evidence that supports a connection between diet and the onset and progress of AD [4,9]. Still, a higher ingestion of DHA may not reflect an increased DHA content in the brain. Valentini et al. [10] observed that the DHA in mice brains did not increase after supplementation with fish oil containing up to 2000 mg/day of DHA (equivalent to a human diet). Also, Yang et al. [11] reported no effect of 6-month supplementation with 300 mg/day DHA on children’s executive functions.

The scientific evidence produced so far shows that the chemical structure of DHA ingested is relevant for more efficient uptake. Moreover, DHA is more bioavailable when it is esterified as phospholipids (PL) as compared to triacylglycerol (TAG), as only PL is able to cross the BBB [12]. In this regard, Nguyen et al. [13] identified the major facilitator superfamily domain-containing protein 2A (Mfsd2a) as the main transporter of DHA across the BBB. The authors also found that Mfsd2a transports DHA specifically in the form of lysophosphatidylcholine (LPC), but not in its free form, which explains the fact that the amount of LPC-DHA accumulates 10-fold higher in the brain when compared with DHA in its free fatty acid form [2]. Such a hypothesis was corroborated by studies in vitro and in vivo carried out by several authors [14,15,16]. More recently, Sugasini et al. [7] also reported that the brains of mice orally treated with LPC-DHA were significantly higher in DHA, in opposition to those supplemented with free DHA, where no differences in DHA content were observed. The same authors also observed that mice supplemented with LPC-DHA had markedly improved spatial learning and memory.

The LPC physiological roles are still controversial because they depend on the type of FA in the acyl chain. While some authors refer to their effect on the upregulation of cholesterol synthesis and a pro-inflammatory effect [17,18], others highlight their anti-inflammatory potential [19,20,21]. The explanation for this relies on the fact that LPC biological activity is acyl chain-dependent. In other words, its pro- or anti-inflammatory effect is related to the acyl chain length and degree of unsaturation [22]. As such, while saturated LPC, containing palmitic acid, is a potent inflammatory mediator, polyunsaturated acyl LPC, like LPC-DHA, can serve as an anti-inflammatory lipid mediator and counteract the inflammation induced by saturated LPC [5,18,22].

Notwithstanding the numerous studies in the literature regarding LPC-DHA, as far as the authors are aware, a nutritional strategy based on food fortification with fish oil extracts enriched in LPC as a way to increase brain cell DHA uptake has never been tested. Indeed, the production of LPC-DHA aiming at oral supplementation, or food fortification, based on the fish oils may find an important application as a way to increase the DHA uptake, especially by the brain cells, and thus contribute to healthier neuronal tissues and higher neutroplasticity. This might be a plausible approach since the PL fraction found in pelagic fish like sardine (*Sardina pilchardus*), chub mackerel (*Scomber colias*) and horse mackerel (*Trachurus trachurus*), depending on the season, contains high levels of DHA, from 36.6 up to 55.1% of total fatty acids (FA) [23,24,25]. 

Atlantic mackerel (*S. scombrus*) is another pelagic fish that is also abundant in the North Atlantic and Mediterranean. Even though it is distributed along the entire Portuguese coast, it is more abundant in the northern region of the country [26]. From a nutritional standpoint, Atlantic mackerel is known to contain high protein content, being a good source of n-3 PUFA and vitamin D as well [27].

Fish by-products like roes, gonads, skin, and heads are also known to contain important amounts of n-3 PUFA, particularly DHA [28]. As these fractions are usually discarded by the fish industry or used in low-value products (as feed, for example), they represent a significant amount of undervalued natural resources that could and should be upgraded through the formulation of added-value products, such as food supplements or ingredients for food fortification [29,30,31].

In view of this, the present work aimed to establish a process for the production of LPC-DHA suitable for supplementation and/or food fortification intended for the prevention of neurodegenerative diseases like AD.

## 2. Results and Discussion

### 2.1. Characterization of Atlantic Mackerel Lipid Fraction

#### 2.1.1. Total Lipids Content

**Figure 1:**
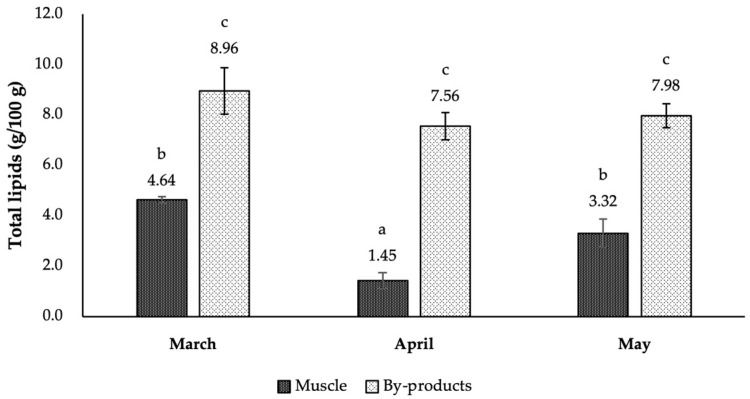
Total lipids content of Atlantic mackerel muscle and by-products captured on the Portuguese coast between March and May 2022 (results are expressed in g/100 g). Error bars correspond to standard deviation. For each variable, different letters correspond to different arithmetic means (*p* < 0.05).

The results concerning the Atlantic mackerel total lipids (TL) content found in Spring/22 (March to May) are shown in Figure 1. Within this period, the fat content determined in muscle samples changed significantly, with the lower contents being determined in April (1.45 g/100 g) and the highest in March (4.64 g/100 g). Similar contents have also been reported by Molversmyr [32] (3.1 g/100 g). Despite this, TL ranging from 4 to 30 g/100 g are usually referred to Atlantic mackerel [33,34]. The lipid composition in pelagic fish like Atlantic mackerel varies seasonally (and even yearly), with regard to multiple external factors including their geographical location (i.e., fishing area), ocean temperatures and food availability [35,36,37]. For example, Guizani and Moujahed [35] observed that the TL contents in Atlantic mackerel captured in the Tunisian northern-east coast were lower during summer months (4.5 g/100 g) and higher in spring (11.1 g/100 g). In turn, Romotowska et al. [36] reported higher fat contents in *S. scombrus* caught during summer (July–September) in Iceland waters, ranging from approximately 20 to 30 g/100 g. Moreover, biological factors, including individuals’ weight, length, maturity stage, spawning, and migration are also known to lead to significant differences in fat contents [37,38]. The Atlantic mackerel spawning season takes place during the winter and spring months, starting in the Mediterranean Sea, after which they migrate to Scotland and the North Sea, where they feed during summer and fall [26,39]. Along the Portuguese coast, it begins in February, yet the peak is likely to occur in March and April [40]. During this period, proteins and lipids are mobilized from the muscle and transferred for gonad production [41]. As such, lower fat contents are typically found during the Atlantic mackerel spawning season. This is in good agreement with our results, where the lowest fat content was determined in fish caught in April.

By-products comprise a very complex group of fractions, whereby their chemical composition can be quite variable [42]. When compared to muscle, data concerning the chemical composition of Atlantic mackerel by-products are scarce. Still, in their study, Setijawati et al. [43] accounted for 3.56 g/100 g of lipids in *S. scombrus* heads. In turn, it is known that the lipid content of the skin of fatty fish, like Atlantic mackerel, may represent more than 50% of fish skin [33]. Also, crude lipid content can vary from less than 5% to 20% in salmon roes [44]. Based on our results, by-product TL content was higher (*p* < 0.05) than those determined in muscle samples, which was somewhat expected since the heads, gonads and skin are tissues typically richer in lipids than the muscle. Our results also show that by-product TL content remained globally unchanged during Spring/22. The highest content was registered in March (8.96 g/100 g), and the lowest in April (7.56 g/100 g). However, several authors have reported seasonal differences in by-product TL content on other species. Despite carrying out in-depth research, no studies were found that presented results for by-products that contained the same organs (heads, skin and gonads). For example, Falch et al. [45] highlighted that differences in cod viscera, TL contents may occur depending on the considered season. Selmi et al. [46] studied the seasonal change of lipid composition of little tuna *Euthynnus alletteratus* by-products and reported higher TL content during winter for the liver and heads, while viscera and gonads tended to present higher amounts in summer. Likewise, Jacobsen et al. [47] observed that liver lipid content and composition from several fish species varied similarly during two consecutive years, with significantly lower values in spring (March, April) and higher values in fall (October, November). Such changes could not be confirmed in this work. One possibility may be related to the fact that the studied period is relatively short in time. Therefore, a longer period should be considered in a near future study, in order to conclude about the seasonal changes in the chemical composition of these by-products.

#### 2.1.2. Fatty Acid Profile

The results regarding the FA profile (relative and absolute content) determined in Atlantic mackerel muscle and by-products are shown in Table 1 and Table 2. While samples obtained from *S. scombrus* captured in March and April showed a profile richer in MUFA (MUFA >> PUFA > SFA), the May counterparts showed a more balanced profile between SFA, MUFA and PUFA. In May, PUFA and SFA relative contents denoted a significant increase, whilst MUFA contents were significantly lower than those determined in March and April. The proportion between these groups is found to be greatly variable among the multiple investigations that have assessed the Atlantic mackerel FA profile [32,34,35,41,48], where factors like the season and geographical location underlie such variability. In this regard, Romotowska et al. [36], for example, identified a significant effect of geographical variation on FA saturation determined in this pelagic fish.

According to our results, MUFA relative contents ranged between 53.9 and 31.8% of total FA, determined both in muscle samples from March and May, respectively. The observed reduction in MUFA was leveraged mainly by 22:1 n-11 (cetoleic acid) and 20:1 n-9 (eicosenoic acid), whose contents have consistently decayed within this period. For example, cetoleic acid relative contents in March corresponded to 20.9% of total FA found in muscle, while in May it represented only 0.8%. Likewise, eicosenoic acid relative contents decreased from 12.3 to 1.8% of total FA determined in muscle. Other MUFA, like oleic acid (18:1 n-9), denoted the opposite trend with significant increments in their relative contents within the studied period. In muscle, these contents increased from 11.3 to 20.1%, while in by-product counterparts they increased from 12.2 to 19.3% of total FA. Among other authors, oleic acid is often presented as the most relevant MUFA in Atlantic mackerel muscle, with approximately 6.0 to 13.8% of total FA [35,36]. However, the relevance of eicosenoic acid is also highlighted by Romotowska et al. [36] and Molversmyr [32]. Such results may be related to the fact that the diet of the Atlantic mackerel mainly consists of copepods, which is considered to be a trophic biomarker for this FA [49]. According to Falk-Petersen et al. [50], 20:1 n-9 and 22:1 n-11 are present in very large amounts in *Calanus* copepods, arising from de novo biosynthesis in these animals. Indeed, the same authors mention that these organisms comprise the major site of the formation of 20:1 n-9 and 22:1 n-11 units in marine food.

Concerning PUFA, the relative content of this group of FA almost doubled in muscle samples reaching a maximum of 39.4% of total FA in May. In any case, the n-3 PUFA has always represented more than 82% of total PUFA determined in Atlantic mackerel samples. These results contributed to the high DHA contents, which were the most abundant PUFA. DHA alone accounted for up to 20.2% of muscle samples from May, where the highest contents were determined. This content is similar to what was determined in previous research including horse mackerel and sardine, still they do not reach those determined in chub mackerel [23,24,25]. Eicosapentaenoic acid (20:5 n-3, EPA) was the second most abundant PUFA, ranging from 4.3 to 9.9% of total FA found in muscle samples from March and May, respectively. This predominance of DHA in Atlantic mackerel, followed by EPA, is also well documented in the available literature [32,34,35,41,48]. 

When compared to MUFA and PUFA, SFA contents varied to a lesser extent. Within this group of FA, palmitic acid (16:0) stood out as the most relevant, representing up to 18.4 and 18.0% of total FA determined in Atlantic mackerel captured in May (muscle and by-products, respectively).

In global terms, the trends observed for SFA, MUFA and PUFA groups in by-products were quite similar to those already described for muscle samples. Indeed, geographical and seasonal differences in terms of FA profile have also been demonstrated for other marine fish by-products, namely those obtained from herring and gadiform species [45,51]. In line with what was described by Aidos et al. [51], our results also denote a wider range of variation in muscle samples than in by-products.

Similar to what was observed for muscle samples, cetoleic (20.9% of total FA), eicosanoic (10.7%) and oleic acids (12.2%) were the most relevant monoenes in March and April, while in May, only oleic acid stood out (19.3%). Once more, this profile may be attributed to this pelagic dietary regime rich in calanoid copepods. Herring by-products also proved to be rich in these FA [51]. The highest PUFA relative contents determined in by-products were also observed in May, where they accounted for 34.9%, while DHA corresponded to 15.8%. In turn, EPA relative contents varied between 4.8 to 9.6% (determined in by-products from April and May, respectively). The obtained fish by-products, for example, from sardine, cod, salmon and tuna, are considered good sources of long-chain n-3 PUFA, having in their composition high levels of DHA and EPA [37]. Rincón-Cervera et al. [52] accounted for 18.0 and 27.5% of DHA in roes and male gonads from Atlantic mackerel, respectively. The same authors also reported EPA relative contents of 7.5 and 11.0% for Atlantic mackerel roes and male gonads. When compared to herring by-products, those from our study showed higher DHA and EPA contents [51]. 

It is worth highlighting the high level of n-3 PUFA, which confers an important anti-inflammatory role in this fish species, proven by the interesting levels of the n-3/n-6 ratio. As AI and TI indices are considered good indicators of the dietary FA influence on CHD, they were also estimated and included in Table 1. Low values of AI (≤0.51) and TI (≤0.30) are recommended for human health [53]. Even though the Atlantic mackerel AI and TI indices were higher than those determined for chub mackerel [25], they were consistently lower than the reference values.

The analysis of the FA absolute content in Atlantic mackerel muscle reinforces that the consumption of this fish species may provide important amounts of key FA (Table 2). For n-3 PUFA, for example, the found contents varied from 248.3 to 937.9 mg/100 g while DHA ranged from 150.9 to 534.2 mg/100 g, in the same order, in the months of April and May. Considering that several studies indicate that a daily intake of 250 mg of DHA has a neuroprotective effect and prevents the onset of AD, such results mean that, in these months, the consumption of approximately 166 to 47 g of Atlantic mackerel will be enough to achieve this beneficial effect. The DHA absolute contents were found to be higher than those reported by Oudiani et al. [41] (60.7–279.3 mg/100 g) for Atlantic mackerel, yet lower than those determined by Molversmyr [32] (735 mg/100 g). Despite this, *S. scombrus* also provides good amounts of other important FA. In the richest month, May, this fish delivers up to 262.7 mg/100 g of EPA.

Furthermore, our results show that Atlantic mackerel by-products also comprise a rich source of n-3 PUFA (up to 2000 mg/100 g), DHA (up to 1000 mg/100 g) as well as linolenic acid (up to 119.5 mg/100 g), showing that this undervalued biomass still encompasses a great upgrading potential, namely for the production of fish oils, for food supplements or food fortification purposes.

#### 2.1.3. Total Lipid Classes

The analysis of the total lipid classes (LC) revealed significant differences (*p* < 0.05) depending on the capture month (Table 3). Moreover, different trends are also observed for muscle and by-products. The higher non-polar lipid (NL) relative contents occurred in the fish captured in March, coinciding with the higher TL content registered. In this month, NL represented nearly 78% of TL found in Atlantic mackerel muscle. Inversely, PL were higher when fish were leaner (in April, during the spawning season), representing 32.20% of TL. The lower PL amount was found in March (25.46% of TL).

The group of NL proved to be consistently higher in by-products, while PL were more abundant in fish muscle. Contrary to what was observed for muscle, the distribution of PL and NL in by-product biomass was not related to the fish fat content. For these, the highest NL relative content was determined in April with 82.7% of TL. Instead, the higher PL were registered in the sub-products of the Atlantic mackerel caught in May.

#### 2.1.4. Fatty Acid Profiles of Phospholipids and Non-Polar Lipid Classes

The FA composition determined for muscle and by-products PL and NL fractions was evaluated and is exhibited in Table 4. Despite the discovered similarities in terms of SFA, significant differences were perceived for MUFA and PUFA groups when muscle and by-product samples were compared. MUFA were more abundant among NL where they accounted for 57.4 and 52.7% of total FA (in muscle and by-products, respectively). These contents were mainly due to three major FA: 22:1 n-11, 20:1 n-9 and 18:1 n-9, whose contents represented between 11.7 and 21.1% of total FA. In organisms, NL acts as energy reserve lipids which are typically rich in MUFA and SFA. Moreover, NL is also closely related to the fish dietary regime. For this reason, a closer look at the NL FA profile may provide important clues regarding the predator–prey relationships and even the feeding habitat. This is possible because specific organisms are known to synthesize specific FA that is incorporated without modification into predators’ NL fraction. This is the case of 22:1 n-11 and 20:1 n-9 which are only biosynthesized de novo by copepods, making such FA a good trophic biomarker for these organisms [54,55]. Their lower contents within the PL fraction suggest that these FA are preferably used for the provision of metabolic energy rather than involved in biomembrane functioning [56]. Likewise, 18:1 n-9 is also considered a trophic marker for copepods, 16:1 n-7 and 20:5 n-3 (EPA) have been linked to diatoms, while DHA (22:6 n-3) was related with dinoflagellates, and 18:1 n-7 with green algae [55]. In turn, other PUFA like 18:4 n-3 and 18:2 n-6 may originate in the ingestion of phytoplankton and benthic organisms, respectively. Non-polar lipids have also shown high amounts of SFA, especially 16:0 and 18:0 (stearic acid). Despite this, SFA’s highest contents were determined in the PL fraction, mainly due to high relative amounts of 16:0 and 18:0. This 16:0 distribution is typical of seafood (as it is typical of membrane lipids in marine organisms [57]), yet it is less usual for 18:0. Even though, similar results can be found in the literature [24,25,58,59] and seems to be related with the FA composition of phosphatidylethanolamine (PE). This might be a plausible explanation for our findings, based on the results regarding PE FA composition (to be discussed in Section 2.2). Concerning PUFA, they were the most relevant group for the PL composition, which was already expected, as PL comprises the group of structural lipids, being major components of cell membranes. Within the polyenes group, DHA stood out with relative contents ranging between 32.9 and 27.6% found in muscle and by-products, respectively. In turn, EPA ranged between 9.3 and 10.1% of total FA. EPA levels are in good agreement with those previously reported for horse mackerel, sardine and chub mackerel [23,24,25]. Still, the found DHA contents proved to be lower. 

These results confirm that both muscle and by-products are good raw materials for obtaining fish oil enriched in PL-DHA. Despite this, for this initial investigation, muscle biomass was used to process this oil, since it delivers higher amounts of PL and DHA. Yet, in the future, for the sake of the sustainable production of such oil, it must rely on by-products or non-tradable species, including those discarded.

### 2.2. Production and Characterization of the Fish Oil Enriched in LPC-DHA

LPC has two distinct isomers that differ only in the positioning of their hydroxyl group within the glycerol backbone: at the *sn-1* position (1-LPC) or *sn-2* position (2-LPC). In fish, long-chain PUFA, like DHA, are mainly located at the PL and TAG *sn-2* position, while MUFA and SFA are usually located at *sn-1* and *sn-3* moiety [60,61,62], and a similar distribution seems to occur in human tissues and plasma [5]. As such, the regioselectivity of the enzymes is a critical factor for the production of LPC-DHA. While phospholipase A_2_ (similar to what occurs in the digestion process) removes dietary DHA found in TAG and PL at *sn-2* position, 1,3-regioselective enzymes like *Thermomyces lanuginosus* and *Rhizomucor miehei* will not.

Considering their relevance as bioactive compounds and their multiple applications, over the years, a great effort has been placed into the optimization of the required methodology for LPC production. Sarney et al. [63], for example, tested the transesterification reaction in a range of alcohols and reaction conditions (time and temperature) using immobilized *R. miehei* lipase. More recently, Mnasri et al. [64] compared multiple enzymes (*R. miehei*, *T. lanuginosus*, *Candida antarctica*) and reaction conditions (substrate ratio, amount of catalyst and temperature), while Yasuda and Yamamoto [65] prepared LPC using lipase B from *C. antarctica* (Novozym ® 435). 

Putting all these together, *R. miehei* was considered to be the best option as it is already widely used by the food industry in multiple biotechnological processes (including lipid processing like alcoholysis reactions and FA esterification) [66].

**Figure 2:**
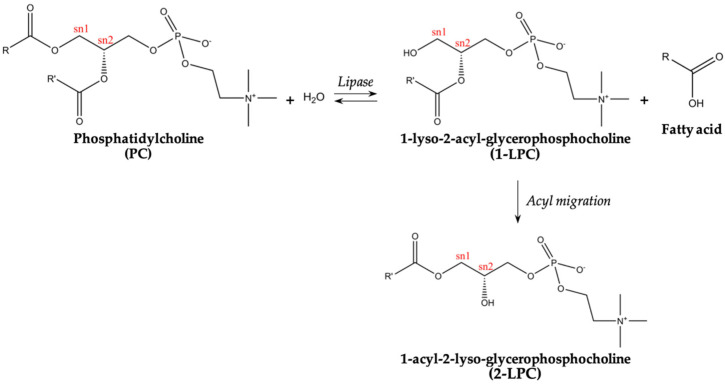
Enzymatic hydrolysis of phosphatidylcholine catalysed by 1,3-regioselective enzyme for the production of lysophosphatidylcholine.

The scheme for this enzymatic reaction is presented in Figure 2. It is worth mentioning that LPC-DHA is resistant to phospholipase A_2_ hydrolysis, being transported and absorbed in this form [67]. 

Apart from the enzyme, the methodology adopted for the PL extraction must also be equated. Chlorinated solvents (including chloroform) are traditionally the first choice to extract lipids contained in a certain matrix. Their toxicity, however, hampers their application to food processing. Hexane is a solvent extensively used by the food industry to extract non-polar compounds like vegetable oils and fats, yet it is not risk-free as well [68]. Since this fish oil is intended to be used as a food supplement and/or for nutraceutical applications, the selected solvents should be restricted to food-grade solvents. Along with this, with the considerable amounts that are expected to be processed and the global potential impact on the environment and occupational health, finding alternative green solvents has become a major concern [69].

In their work, Breil et al. [70] concluded that ethyl acetate could be good a candidate to replace hexane for salmon lipids extraction as this solvent proved to be effective for TAG extraction. However, due to its low polarity, only a small fraction of PL could be recovered with ethyl acetate. As such, this solvent might be a suitable option whenever the relative abundance of TAG, PL or other lipid classes is not relevant. In order to be more selective for polar compounds like PL, the use of more polar solvents was required. In this regard, the investigation of Breil et al. [70] has also highlighted that, among the multiple tested solvents, only isopropanol and ethanol were able to extract both PC and PE, the latter being the most efficient one. Taking this into consideration, ethyl acetate and ethanol were selected to extract the lipid fraction in our experiments, as they are included on the US Food and Drug Administration’s Generally Recognized As Safe list for use in foods and pharmaceuticals [71]. For that, the fish oil enriched in LPC-DHA was obtained by a two-stage extraction process, combining ethyl acetate and ethanol. At first, the fish biomass was extracted using ethyl acetate, in order to remove an important fraction of NL (especially TAG that are present in high amounts and could interfere with the yield of PL conversion into LPC). In a second moment, the resulting biomass was extracted once more with ethanol. This methodology allowed us to obtain an extract rich in PL with lower contents of interfering TAG, as is shown in Table 5. In this extract (designated as PreH_extract) PL accounted for 50.79% of TL classes, which means an increment of nearly 30% when compared to PL determined in the raw matter (where PL represented only 22.46%). Among these, PC corresponded to 57.76% of PL present determined in PreH_extract, while PE represented 42.24% (Table 6). In turn, NL corresponded to 49.21% of TL classes, being TAG the most abundant, representing 30.76%.

The PreH_extract underwent enzymatic hydrolysis with Lipozyme® RM. The resulting extract (PostH_extract) was also studied for its LC and PL classes (Table 5 and Table 6). In line with what was expected, the PL hydrolysis led to a significant increase in NL fraction and the concomitant decrease in PL. During this enzymatic reaction, the deacylation of PL takes place, i.e., one acyl chain is released from the glycerol backbone as a free fatty acid (FFA). With this hydrolysis reaction, the FFA contents increased from 11.50 to 46.04% of TL. On the other hand, both 1,3 DAG and TAG remained unchanged, showing that Lipozyme® RM was more specific for PL deacylation.

A closer look into the PL classes composition (Table 6) allows us to conclude that 43.61% of the existing PC was converted into LPC. In this regard, other authors, like Haraldsson and Thorarensen [72] and Haas et al. [73], have also attained comparable yields for PC hydrolysis. In Haas et al. [73] experiments, using hexane as the main solvent, and in the presence of calcium chloride, the authors determined a yield of about 40%. These results proved to be worse than those determined by Sarney et al. [63] and Yang et al. [74] who evaluated the LPC production via ethanolysis of PC. In both studies, this enzymatic reaction rendered between 80 to 95% of LPC conversion. The results (Table 6) also demonstrate that part of the PE was hydrolysed as well (35.3%), even though to a lower extent than PC. In the end, in the PostH_extract, LPC and LPE corresponded to 25.19 and 14.90% of total PL, respectively. Although Haas et al. [73] concluded that PC and PE were hydrolyzed at essentially equal rates, these findings are in good agreement with what was reported by Ono et al. [75]. According to these authors, the partial hydrolysis of egg lecithin with immobilized *R. miehei* resulted in 34.7% of LPC and 13.9% of LPE. The fact that egg PC has shorter-chain fatty acids than those present in our PL extracts, and that lipase from *R. mihei* seems to be more active over short-chain fatty acids, might explain the lower hydrolysis rates found in our results [76,77]. Despite this, our results show that there is still a great potential for improvement in terms of LPC and/or LPE production. In regard to this, this optimization process is already being carried out, considering the multiplicity of factors that might positively or negatively impact enzyme activity. An adequate amount of water, for example, promotes the enzyme’s molecular flexibility, which is critical to maintaining the enzyme’s adequate hydration and maximum activity [22,75]. Likewise, lipase from *R. mihei*, is known to be quite active and stable in systems with low water activity [76]. Along with the water amount, the selected solvent also impacts lipase activity. While Haraldsson and Thorarensen [72] found that the reaction rate was solvent-dependent and inversely proportional to the solvent polarity (fastest in hexane, slower in toluene and slowest in ethyl acetate), Yang et al. [74] reported high hydrolysis rates in a solvent-free media.

Concerning TAG, Haas et al. [73] found that they were rapidly and completely hydrolysed, while PC and PE were hydrolysed at a lower rate. Yet, a different scenario is exposed in the results shown in Table 5, where the hydrolysis of this class of lipids seemed to be nonexistent. Based on this, it might be hypothesized that the FA located at the TAG *sn*-*1* position might not be hydrolyzed by this enzyme, which can make sense if we consider the enzyme specificity for a certain FA.

The assessment of the FA profile in these PL fractions (as shown in Table 7) has also highlighted significant changes between the extracts before and after the hydrolysis reaction. Indeed, significant differences arose from the comparison of the PC and PE FA profile from PreH_extract with the PC, LPC, PE and LPE found in PostH_extract. Such changes were particularly relevant for DHA, with its relative content significantly concentrated both in hydrolysed and non-hydrolysed PL forms. When compared to PC and PE initial content (33.6% and 41.4%, respectively, found in PreH_extract), DHA more than doubled. In PostH_extract, this FA represented, in the same order, 73.6% and 73.4% of the total FA determined in LPC and LPE fractions. For LPC, these results represent a concentration of 120%, when compared to PC initial contents. DHA contents were significantly concentrated in non-hydrolysed forms as well, accounting for 55.6 and 49.9% of total FA determined in PC and PE from PostH_extract.

As the production of LPC enriched in DHA was the main goal of this research work, such results turned out to be promising, even when compared to other works. Ono et al. [75] reported a DHA concentration of nearly 13% in their experiments, from 11.5 to 24.6% in the hydrolysate obtained from egg lecithin, and from 35.6 to 48.0% when squid lecithin was used as substrate.

Interestingly, the *R. miehei* lipase seemed to act differently in other PUFA as they evolved in distinct manners. The EPA relative content, for example, remained unchanged for PC found in PreH_extract and PC and LPC present in PostH_extract (12.0, 9.4 and 10.9%, respectively). Nevertheless, the enzyme seemed to be more active over the EPA esterified to PE in PreH_extract (9.2%), resulting in a significant reduction in this FA both in PE and LPE from PostH_extract, that accounted for, in the same order, 6.8 and 3.6% of total FA. In turn, for docosapentaenoic acid (22:5 n-3, DPA), a significant decrease was only observed in hydrolysed forms of LPC and LPE (1.4 and 0.8%, respectively), while linolenic acid (18:2 n-6, LA) was almost inexistent in PL fractions from PostH_extract. Despite this, at the end of the enzymatic hydrolysis, the sum of all PUFA reached relative contents as high as 94.1% of total FA in LPC. Likewise, in PostH_extract, the polyenoic group accounted for 86.6% of LPE, 78.3% in PC and 73.1% in PE. The n-3 PUFA alone comprised between 69.6 to 89.1% of total FA determined in PL fractions from PostH_extract.

The enzymatic hydrolysis strongly affected the SFA and MUFA relative contents as well. For SFA, its initial levels of 28.3 and 19.7% (PC and PE in PreH_extract, respectively) were significantly decreased up to 1.6 to 9.1% (PE and LPC found in PreH_extract). Among these groups of FA, palmitic acid (16:0) was the preferential target of *R. miehei* lipase. Despite its relatively high content in PC and PE from PreH_extract (being the second most abundant FA following DHA), after the enzymatic hydrolysis, only low amounts of palmitic acid were detected (below 1.5%), both in non-hydrolysed and hydrolysed PL. Among MUFA, 20:1 n-9, with maximum contents of 0.6%, was almost inexistent in PL fractions present in hydrolysed extract as well. The same stands for 18:1 n-9, with 0.7 and 1.1% of total FA found in LPC and LPE, despite its higher contents in non-hydrolysed forms present in the PostH_extract that accounted for nearly 3%.

Putting all these together, the results provide important clues about the *R. miehei* lipase action over the PL extracts, where it seemed to release specific FA. These might be related to two main factors: (1) the position of the FA in the glycerol backbone and (2) the specificity of the lipase for determined FA. The fact that DHA is predominantly found in the *sn-2* position of fish and seafood lipids [5] prevented it from being hydrolysed by regioselective enzymes, as occurs with *R. miehei* lipase. Instead, the FA that are mainly esterified at the PL *sn-1* position, like SFA, are more susceptible to their action, which might explain the extensive hydrolysis of palmitic acid (16:0) both in PC and PE. In their work, Kotogan et al. [66] and her co-workers found that the type and amount of released FA were different depending on the source of the oil. For rapeseed oil, these authors reported a significant increment of unsaturated FA like oleic (18:1 n-9), linoleic (18:2 n-6) and linolenic acids (18:3 n-3), without remarkable changes in SFA contents. A different scenario was observed for linseed oil, where both SFA and unsaturated FA were released. In turn, for grapeseed oil, palmitic acid (16:0) was the predominant FA released [66]. Likewise, when the lipase from *R. miehei* was used to produce TAG enriched with PUFA throughout an esterification reaction, Rivero-Pino et al. [78] reported that this enzyme initially esterifies stearic acid (18:0), while linolenic acid (18:3 n-3) is preferably esterified in the last step of the esterification, suggesting that it has a slight selectivity toward unsaturated FA.

Lipases are globally known to preferentially act on SFA and MUFA over n-3 PUFA. This relies on the fact that the double bonds in the latter cause steric hindrance in the active site of a lipase [79], slowing down its action over FA. Moreover, our results also show that a portion of the EPA esterified to PC and PE present in fish oil was hydrolysed as well. Even though this is not desirable, a significant preference of this enzyme for EPA over DHA was already reported by other researchers [80,81,82]. Concerning this, Bispo et al. [80] recall that the proximity of the double bond to the carboxylic group makes it difficult for the substrate to adapt to the lipase active center. As for EPA and DHA, concerning the first double bond found in positions 5 and 4, respectively, the former will be more easily hydrolysed. Based on this, even if DHA undergoes acyl migration within the glycerol backbone, it might not be hydrolysed.

## 3. Materials and Methods

### 3.1. Material and Reagents

Chloroform, methanol, ethyl acetate, acetone, absolute ethanol and hexane were purchased from Carl Roth; diethyl ether, acetic acid, acetyl chloride, n-heptane and ammonia 25% were from Panreac, while phosphomolybdic acid, anhydrous sodium sulphate, copper sulphate (II) were purchased from Merck. Ultrapure water was obtained from a Millipore Milli-Q water purification system.

The experiments were carried out using Atlantic mackerel (*S. scombrus*). Ten individuals, captured on the Portuguese coast, were purchased monthly from a local market nearby Lisbon, in March, April and May/2022. The fish was transported to the laboratory under refrigeration, in cool boxes. Once samples arrived at the laboratory, they were immediately processed. For that, the viscera and bones were removed and discarded and the muscle separated from the by-products fraction. For the later fraction head, skin and gonads (male and female) were pooled. Both muscle and by-products were minced (Grindomix GM 200, Dusseldorf, Germany), vacuum-sealed in a plastic bag and stored at −80 °C.

### 3.2. DHA-Rich Phospholipids Fish Oil Production

The fish oil production was carried out following a 2-step process with batches of approximately 500 g of homogenised muscle from Atlantic mackerel. 

The fish captured in March/22 was used for the DHA-rich phospholipids fish oil production once these samples present higher total lipids (TL) content and those individuals were bigger in size, thus providing a greater amount of biomass to work with. Firstly, the TAG fraction was extracted with ethyl acetate in a proportion of 1:4 (fish:ethyl acetate, *w*/*v*) using a Polytron (Kinematica, Switzerland). The homogenate was then centrifuged under refrigeration (4 °C) at 3975× *g* for 10 min. To obtain de PL, this process was repeated once more, extracting the remaining biomass with the same volume of absolute ethanol. The solvent was then evaporated and the residue resuspended in hexane at a final concentration of 47.5 mg/mL (PreH_extract).

On the eve of hydrolysis, 250 mg of the immobilized enzyme (Lipozyme® RM, 275 IUN/g obtained from Novozymes) was weighed for a test tube, hydrated with water (50% *w*/*w*) and left overnight at room temperature, protected from light. Since this lipase is very active and stable in systems with low water activity [76], this is a mandatory step. Then, 1.5 mL of PreH_extract was added, and the test tube’s atmosphere was filled with nitrogen and left to react for 24 h at 40 °C. After this period, the hydrolysed extract (PostH_extract) was collected. The test tube and enzyme were then washed with 1 mL of hexane and 1 mL of ethanol. These fractions were then combined in the same test tube and stored at −80 °C until further analysis.

### 3.3. Total Lipids Content

The Atlantic mackerel TL content (muscle and by-products) was determined gravimetrically according to the Bligh and Dyer [83] method.

### 3.4. Lipid Classes Profile

#### 3.4.1. Total Lipid Classes

The assessment of total lipid classes distribution was accomplished following the analytical protocol described in Bandarra et al. [24] by means of high-performance thin-layer chromatography (HPTLC). Four microliters of lipids extracts (at 10 mg/mL) were loaded onto a Silica gel 60 F₂₅₄ HPTLC (200 × 100 mm, 0.5 mm) plate (Merck, Germany) with the Linomat 5 (CAMAG, Switzerland), eluted with n-hexane:diethyl ether:acetic acid (65:35:1 *v*/*v*/*v*), and immersed in a 5% (*w*/*v*) solution of phosphomolybdic acid in ethanol. After staining, plates were left in the oven at 100 °C for 1 h and band quantification was performed by densitometry TLC Scanner 4 (CAMAG, Muttenz, Switzerland) set to 650 nm (using the tungsten lamp).

#### 3.4.2. Polar Lipid Classes

The PL profile was also determined by HPTLC following the method described by Handloser et al. [84]. Four microliters of lipids extracts (at 10 mg/mL) were loaded onto a Silica gel 60 F₂₅₄ HPTLC plate with the Linomat 5, eluted with a mixture of chloroform:methanol:water:ammonia 25% (60:34:4:2 *v*/*v*/*v*/*v*). The plate was derivatized with a solution of copper sulphate (II) and the bands were revealed in an oven set to 140 °C for 30 minutes. A densitogram was obtained in a TLC Scanner adjusted to 360 nm (deuterium lamp).

### 3.5. Fatty Acid Profile 

The FA profile was determined by acid-catalysed transesterification as described by Bandarra et al. [23]. Briefly, 300 mg of freeze-dried edible muscle was weighed to a test tube and 5 mL of a 5% acetyl chloride-methanolic solution (freshly prepared before use) was added. Tubes were left to react for 1 h in a water bath adjusted to 80 °C. Once sample extracts were cooled, 1 mL of Milli-Q water and 2 mL n-heptane were added. Then, tubes were agitated and centrifuged for 3 minutes at 3000× *g*. The organic phase was then collected and filtered through anhydrous sodium sulphate. The final extract was then analysed by gas chromatography, in a Scion 456-GC gas chromatograph (West Lothian, UK), equipped with a capillary column DB-WAX (Agilent Technologies, Santa Clara, CA, USA) (film thickness, 0.25 μm), 30 m × 0.25 mm i.d. The separation of the FAME was carried out with helium as the carrier gas, using a temperature program for the column starting at 180 °C for 5 minutes, increasing to 220 °C at 4 °C/min and holding for 24 min. The FA identification was based on their retention time, using a standard mix (PUFA-3, Menhaden oil, Sigma-Aldrich) as a reference. Results were expressed both as a percentage of its relative content, as well as in mg/100 g of edible part on a wet weight basis. The absolute content was estimated using the corrective factor proposed by Weihrauch et al. [85]. To evaluate the tendency of Atlantic mackerel to influence the incidence of coronary heart disease (CHD), the atherogenic (AI) and thrombogenic (TI) indices were calculated according to the following formulas proposed by [86]: AI = ([12:0] + 4 × [14:0] + [16:0])/([n-6 PUFA] + [n-3 PUFA] + [MUFA]); TI = ([14:0] + [16:0] + [18:0])/(0.5 × [MUFA] + 0.5 × [n-6 PUFA] + 3 × [n-3 PUFA] + [n-3 PUFA]/[n-6 PUFA]).

### 3.6. Fatty Acid Profile of Lipid Classes

#### 3.6.1. Fish Total Lipid Classes

The FA analyses of PL and NL (i.e., phospholipids and TAG, respectively), were performed only in fish samples, caught in March/22. As such, fish total lipid extracts (50 mg/mL) were fractionated by preparative TLC (Silica gel 60 F₂₅₄, 2 mm from Merck, Germany), using the same elution mixture mentioned above. Instead of phosphomolybdic acid, plates were sprayed with 0.2% dichlorofluorescein in ethanol, and visualized under UV light at 254 nm. The silica portions containing the lipid fractions were scratched from the plate and the FAME were determined as described earlier.

#### 3.6.2. DHA-Rich Fish Oil Polar Lipid Classes

The FA analysis of PL classes found in PreH_extracts and PostH_extracts was performed as well. For this, a previous fractionation by means of SPE was carried out in order to remove other interfering lipid classes. For this, Silica Isolute® 500 mg columns were wet with 3 mL of chloroform, after which the extracts were applied. Non-polar lipids (TAG) were eluted with 40 mL of chloroform, lipids with intermediate polarity (like diacylglycerols (DAG), cholesterol (CH), and FFA) were eluted with 20 mL of acetone:methanol (9:1 *v*/*v*), while PL were eluted with 20 mL of methanol. The solvent in the PL fraction was then evaporated and resuspended in chloroform at 50 mg/mL. The extracts were applied with Linomat 5 in Silica gel 60 F₂₅₄ HPTLC plates and eluted with a mixture of chloroform:methanol:water:ammonia 25% (60:34:4:2 *v*/*v*/*v*/*v*). For the band’s identification, a small portion of the plate was cut (Smart Cut, CAMAG, Muttenz, Switzerland) and derivatized with copper sulphate (II) as previously described. The silica portions containing the lipid fractions (PC, PE, LPC and LPE) were scratched from the plate and the FAME was determined as described earlier.

### 3.7. Statistical Analysis

The data were tested for normality and the homogeneity of variance using Kolmogorov–Smirnov’s test and Levene’s *F*-test, respectively. Since these parameters were confirmed, analysis of variance One-Way (for fish oil samples) and Factorial (fish muscle and by-product samples) ANOVA using Statistica^TM^ v.12 software (StatSoft Inc, Palo Alto, CA, USA) were carried out. Means were compared and significant differences (*p* < 0.05) were determined according to Tukey’s honest significant differences (HSD) test.

## 4. Conclusions

The approach proposed in this research allowed the production of DHA-LPC extracts with 27% less SFA, 10% less MUFA, 40% more PUFA and 40% more DHA. These are promising results in the context of a future nutritional strategy aiming at increased brain DHA uptake, intended to prevent the onset of neurological diseases like AD. As such, further studies focused on the application of these extracts as oral supplements or via food fortification should be considered.

Apart from the health benefits, the production of these DHA-LPC extracts might uncover an opportunity to upgrade low-value species (like Atlantic and Chub mackerel), as well as fish by-products (including the skin, heads and gonads), making a more sustainable use of the existing marine resources. The proposed methodology could also find other applications, such as the production of fish oils with increased n-3 PUFA levels.

## Figures and Tables

**Table 1:** Fatty acid relative content of Atlantic mackerel muscle and by-products between March and May 2022 (expressed in % of total fatty acid).

	March		April		May	
	Muscle	By-products	Muscle	By-products	Muscle	By-products
14:0	4.6 ± 0.3 ^b,c^	5.3 ± 0.3^c^	3.1 ± 0.6 ^a^	4.1 ± 0.1 ^b^	2.5 ± 0.2 ^a^	3.1 ± 0.1 ^a^
16:0	11.5 ± 0.4 ^a^	12.5 ± 0.1 ^a,b^	13.7 ± 1.1 ^b,c^	14.8 ± 0.3 ^c^	18.4 ± 0.3 ^d^	18.0 ± 0.3 ^d^
18:0	2.8 ± 0.1 ^b^	2.4 ± 0.1 ^a^	4.6 ± 0.2 ^d^	3.4 ± 0.1 ^c^	5.4 ± 0.1 ^e^	4.6 ± 0.1 ^d^
**SFA**	**20.7 ± 0.8 ^a^**	**22.2 ± 0.4 ^a,b^**	**23.1 ± 1.7 ^b^**	**24.2 ± 0.2 ^b^**	**27.7 ± 0.5 ^c^**	**27.6 ± 0.3 ^c^**
16:1 n-7	2.7 ± 0.1 ^b^	3.6 ± 0.1 ^c^	2.1 ± 0.3 ^a^	3.3 ± 0.1 ^c^	3.6 ± 0.2 ^c^	4.1 ± 0.1 ^d^
18:1 n-9	11.3 ± 0.2 ^a^	12.2 ± 0.2 ^a,b^	12.7 ± 0.7 ^b^	16.1 ± 0.2 ^c^	20.1 ± 0.2 ^d^	19.3 ± 0.1 ^d^
18:1 n-7	2.1 ± 0.1 ^a^	2.0 ± 0.1 ^a^	2.6 ± 0.1 ^b^	2.6 ± 0.1 ^b^	3.9 ± 0.4 ^c^	3.9 ± 0.1 ^c^
20:1 n-9	12.3 ± 0.2 ^f^	10.7 ± 0.2 ^e^	9.6 ± 0.7 ^d^	8.6 ± 0.2 ^c^	1.8 ± 0.1 ^a^	2.8 ± 0.1 ^b^
22:1 n-11	20.9 ± 0.6 ^c^	15.7 ± 0.4 ^b^	15.5 ± 3.3 ^b^	14.2 ± 0.3 ^b^	0.8 ± 0.1 ^a^	3.0 ± 0.4 ^a^
22:1 n-9	1.4 ± 0.0 ^c^	0.9 ± 0.1 ^a,b,c^	1.0 ± 0.6 ^a,b,c^	1.1 ± 0.0 ^b,c^	0.4 ± 0.1 ^a^	0.5 ± 0.1 ^a,b^
24:1 n-9	1.6 ± 0.1 ^b,c^	1.2 ± 0.1 ^a,b,c^	1.6 ± 0.6 ^b,c^	1.6 ± 0.1 ^c^	0.8 ± 0.0 ^a^	0.9 ± 0.1 ^a,b^
**MUFA**	**53.9 ± 0.7 ^c^**	**48.0 ± 0.4 ^b^**	**46.7 ± 4.1 ^b^**	**49.9 ± 0.4 ^b,c^**	**31.8 ± 0.2 ^a^**	**35.8 ± 0.5 ^a^**
18:2 n-6	1.7 ± 0.0 ^b^	1.6 ± 0.0 ^a,b^	2.1 ± 0.3 ^c^	1.9 ± 0.0 ^b,c^	1.3 ± 0.1 ^a^	1.3 ± 0.1 ^a^
18:3 n-3	0.9 ± 0.0 ^c^	1.2 ± 0.0 ^d^	0.7 ± 0.1 ^a^	0.8 ± 0.0 ^b,c^	0.8 ± 0.1 ^a,b^	0.8 ± 0.1 ^b,c^
18:4 n-3	2.2 ± 0.1 ^d^	3.1 ± 0.1 ^e^	1.1 ± 0.2 ^a^	1.7 ± 0.1 ^c^	1.4 ± 0.1 ^b^	1.4 ± 0.0 ^b^
20:4 n-6	0.7 ± 0.0 ^a^	0.7 ± 0.0 ^a^	1.0 ± 0.1 ^b^	0.9 ± 0.1 ^a,b^	1.2 ± 0.1 ^c^	1.2 ± 0.1 ^c^
20:5 n-3	4.3 ± 0.0 ^a^	5.6 ± 0.1 ^b^	4.9 ± 0.8 ^a,b^	4.8 ± 0.2 ^a,b^	9.9 ± 0.6 ^c^	9.6 ± 0.2 ^c^
22:5 n-3	1.3 ± 0.0 ^a^	1.3 ± 0.0 ^a^	1.4 ± 0.1 ^a^	1.3 ± 0.1 ^a^	1.9 ± 0.1 ^b^	1.8 ± 0.1 ^b^
22:6 n-3	8.8 ± 0.1 ^a^	9.7 ± 0.1 ^a^	14.2 ± 2.0 ^b^	8.8 ± 0.3 ^a^	20.2 ± 1.2 ^c^	15.8 ± 0.2 ^b^
**PUFA**	**22.6 ± 0.2 ^a^**	**26.1 ± 0.3 ^a,b^**	**28.1 ± 3.5 ^b^**	**22.9 ± 0.5 ^a^**	**39.4 ± 1.5 ^d^**	**34.9 ± 0.4 ^c^**
**n-3 PUFA**	**18.9 ± 0.2 ^a^**	**22.6 ± 0.3 ^a,b^**	**23.4 ± 3.1 ^b^**	**18.8 ± 0.5 ^a^**	**35.5 ± 1.6 ^d^**	**30.9 ± 0.3 ^c^**
**n-6 PUFA**	**3.3 ± 0.0 ^a^**	**3.3 ± 0.0 ^a^**	**4.3 ± 0.4 ^c^**	**3.8 ± 0.0 ^b^**	**3.5 ± 0.1 ^a,b^**	**3.7 ± 0.1 ^a,b^**
**n-3/n-6**	**5.8 ± 0.1 ^a^**	**6.9 ± 0.1 ^b^**	**5.4 ± 0.4 ^a^**	**4.9 ± 0.1 ^a^**	**10.1 ± 0.8 ^d^**	**8.4 ± 0.3 ^c^**
**AI**	**0.4**	**0.5**	**0.4**	**0.4**	**0.4**	**0.4**
**TI**	**0.2**	**0.2**	**0.2**	**0.3**	**0.2**	**0.2**

Values are presented as the average ± standard deviation. In the same line, different letters represent significantly different arithmetic means (*p* < 0.05). AI, atherogenic index; TI, thrombogenic index.

**Table 2:** Fatty acid absolute content of Atlantic mackerel muscle and by-products between March and May 2022 (expressed in mg/100 g of wet weight).

	March		April		May	
	Muscle	By-products	Muscle	By-products	Muscle	By-products
14:0	176.2 ± 12.3 ^c^	385.2 ± 16.0 ^e^	32.9 ± 6.4 ^a^	255.3 ± 7.0 ^d^	65.6 ± 4.6 ^b^	194.5 ± 2.7 ^c^
16:0	435.9 ± 14.3 ^b^	910.4 ± 10.0 ^d^	145.8 ± 12.2 ^a^	923.8 ± 14.6 ^d^	485.9 ± 9.0 ^c^	1112.3 ± 15.1 ^e^
18:0	103.9 ± 0.8 ^b^	179.0 ± 2.1 ^d^	48.9 ± 2.2 ^a^	214.7 ± 1.8 ^e^	142.6 ± 2.4 ^c^	282.5 ± 5.6 ^f^
**SFA**	**782.4 ± 29.9 ^b^**	**1616.2 ± 23.7 ^d^**	**246.2 ± 17.7 ^a^**	**1505.6 ± 9.9 ^c^**	**729.5 ± 12.6 ^b^**	**1697.8 ± 17.3 ^e^**
16:1 n-7	100.5 ± 3.2 ^b^	262.3 ± 4.3 ^e^	22.9 ± 3.2 ^a^	205.4 ± 5.3 ^c^	94.3 ± 3.4 ^b^	249.6 ± 6.1 ^d^
18:1 n-9	426.6 ± 5.7 ^b^	886.8 ± 7.9 ^d^	134.9 ± 7.7 ^a^	1004.8 ± 14.9 ^e^	530.8 ± 4.1 ^c^	1192.7 ± 3.9 ^f^
18:1 n-7	77.6 ± 0.7 ^b^	148.6 ± 1.5 ^d^	27.6 ± 1.4 ^a^	159.0 ± 0.2 ^d^	102.5 ± 9.4 ^c^	242.1 ± 2.8 ^e^
20:1 n-9	461.9 ± 5.5 ^d^	780.3 ± 9.9 ^f^	101.5 ± 7.6 ^b^	537.0 ± 10.2 ^e^	48.4 ± 0.4 ^a^	174.6 ± 9.0 ^c^
22:1 n-11	789.7 ± 23.3 ^c^	1143.0 ± 29.7 ^e^	164.8 ± 35.6 ^b^	883.4 ± 17.4 ^d^	20.1 ± 1.1 ^a^	186.0 ± 22.3 ^b^
24:1 n-9	58.4 ± 2.6 ^b^	91.4 ± 3.0 ^c^	16.7 ± 6.7 ^a^	102.6 ± 3.4 ^c^	21.1 ± 1.0 ^a^	53.6 ± 5.6 ^b^
**MUFA**	**2033.8 ± 25.2 ^c^**	**3496.0 ± 32.3 ^f^**	**496.9 ± 43.9 ^a^**	**3107.0 ± 23.5 ^e^**	**839.0 ± 5.3 ^b^**	**2203.7 ± 32.8 ^d^**
18:2 n-6	63.0 ± 0.8 ^c^	116.6 ± 1.7 ^e^	22.3 ± 2.5 ^a^	119.5 ± 2.0 ^e^	35.1 ± 1.1 ^b^	82.9 ± 2.7 ^d^
18:3 n-3	33.1 ± 0.7 ^c^	85.5 ± 1.3 ^e^	6.9 ± 0.7 ^a^	50.2 ± 0.9 ^d^	20.2 ± 0.7 ^b^	49.8 ± 3.1 ^d^
18:4 n-3	84.4 ± 1.5 ^c^	224.2 ± 4.2 ^e^	11.6 ± 1.5 ^a^	108.4 ± 3.6 ^d^	36.4 ± 2.1 ^b^	86.9 ± 1.7 ^c^
20:4 n-6	25.8 ± 1.4 ^b^	49.5 ± 0.8 ^d^	10.8 ± 1.1 ^a^	53.7 ± 1.2 ^d^	31.2 ± 1.7 ^c^	78.3 ± 3.3 ^e^
20:5 n-3	161.5 ± 0.5 ^b^	410.7 ± 6.8 ^e^	52.3 ± 8.1 ^a^	300.4 ± 8.0 ^d^	262.7 ± 15.6 ^c^	588.4 ± 12.2 ^f^
22:5 n-3	49.9 ± 0.8 ^b^	97.4 ± 0.6 ^d^	14.2 ± 1.3 ^a^	77.5 ± 1.1 ^c^	50.7 ± 2.4 ^b^	110.6 ± 4.4 ^e^
22:6 n-3	333.5 ± 4.5 ^b^	706.9 ± 8.6 ^d^	150.9 ± 21.0 ^a^	546.1 ± 14.9 ^c^	534.2 ± 31.1 ^c^	975.5 ± 11.5 ^e^
**PUFA**	**851.7 ± 7.8 ^b^**	**1904.9 ± 26.5 ^e^**	**299.5 ± 37.1 ^a^**	**1409.5 ± 31.1 ^d^**	**1040.6 ± 40.1 ^c^**	**2134.2 ± 25.3 ^f^**
**n-3 PUFA**	713.1 ± 7.2 ^b^	1646.1 ± 24.1 ^e^	248.3 ± 33.3 ^a^	1170.6 ± 28.7 ^d^	937.9 ± 42.9 ^c^	1903.2 ± 21.9 ^f^
**n-6 PUFA**	124.0 ± 1.2 ^c^	240.0 ± 2.2 ^e^	45.9 ± 4.0 ^a^	236.5 ± 2.0 ^e^	92.8 ± 3.7 ^b^	225.6 ± 6.8 ^d^

Values are presented as the average ± standard deviation. In the same line, different letters represent significantly different arithmetic means (*p* < 0.05).

**Table 3:** Lipid classes profile of Atlantic mackerel muscle and by-products between March and May 2022 (expressed in % of total lipid content).

	March		April		May	
	Muscle	By-products	Muscle	By-products	Muscle	By-products
PL	22.46 ± 1.07 ^b,c^	19.07 ± 3.93 ^a,b^	32.20 ± 2.43 ^e^	17.29 ± 0.61 ^a^	26.95 ± 4.13 ^d^	23.29 ± 0.63 ^c^
NL	77.54 ± 1.07 ^c,d^	80.93 ± 3.93 ^d,e^	67.88 ± 2.43 ^a^	82.71 ± 0.61 ^e^	73.05 ± 4.13 ^b^	76.71 ± 0.63 ^c^

Values are presented as the average ± standard deviation. In the same line, different letters represent significantly different arithmetic means (*p* < 0.05). PL—phospholipids; NL—non-polar lipids.

**Table 4:** Fatty acid profile in PL and TAG fractions of Atlantic mackerel (captured in March) muscle and by-products (expressed in % of total fatty acid).

	Muscle		By-products	
	PL	TAG	PL	TAG
14:0	1.0 ± 0.1 ^a^	6.1 ± 0.2 ^b^	1.1 ± 0.2 ^a^	5.9 ± 0.1 ^b^
16:0	16.6 ± 0.5 ^b^	12.3 ± 0.3 ^a^	17.8 ± 1.0 ^b^	13.1 ± 0.0 ^a^
18:0	8.1 ± 0.5 ^b^	2.3 ± 0.0 ^a^	7.8 ± 0.2 ^b^	2.4 ± 0.0 ^a^
**SFA**	**27.1 ± 1.1 ^b^**	**23.0 ± 0.5 ^a^**	**28.4 ± 1.3 ^b^**	**23.2 ± 0.1 ^a^**
16:1 n-7	0.8 ± 0 ^a^	3.3 ± 0.1 ^c^	1.4 ± 0.1 ^b^	3.9 ± 0.0 ^d^
18:1 n-9	5.0 ± 0.1 ^a^	13.2 ± 0.2 ^d^	9.2 ± 0.2 ^b^	12.6 ± 0.2 ^c^
18:1 n-7	2.6 ± 0.0 ^b^	2.1 ± 0.1 ^a^	2.2 ± 0.1 ^a^	2.2 ± 0.1 ^a^
20:1 n-9	3.3 ± 0.1 ^b^	13.3 ± 0.2 ^d^	2.6 ± 0.2 ^a^	11.7 ± 0.2 ^c^
22:1 n-11	1.2 ± 0.1 ^a^	21.1 ± 1 ^c^	1.4 ± 0.3 ^a^	18.4 ± 0.6 ^b^
24:1 n-9	1.3 ± 0.0 ^a^	1.3 ± 0.1 ^a^	2.0 ± 0.3 ^b^	1.3 ± 0.1 ^a^
**MUFA**	**15.2 ± 0.3 ^a^**	**57.4 ± 1 ^d^**	**20.1 ± 0.7 ^b^**	**52.7 ± 0.7 ^c^**
18:2 n-6	2.3 ± 0.0 ^c^	1.7 ± 0.1 ^b^	1.4 ± 0.1 ^a^	1.6 ± 0.0 ^b^
18:3 n-3	0.8 ± 0.0 ^b^	0.9 ± 0.1 ^b^	0.5 ± 0.0 ^a^	1.1 ± 0.0 ^c^
18:4 n-3	0.6 ± 0.0 ^a^	2.6 ± 0.2 ^b^	0.6 ± 0.0 ^a^	3.0 ± 0.1 ^c^
20:4 n-6	1.6 ± 0.0 ^b^	0.5 ± 0.0 ^a^	1.7 ± 0.1 ^c^	0.5 ± 0.0 ^a^
20:5 n-3	10.1 ± 0.3 ^d^	3.3 ± 0.3 ^a^	9.3 ± 0.3 ^c^	4.6 ± 0.2 ^b^
22:5 n-3	1.9 ± 0.0 ^b^	1.1 ± 0.1 ^a^	1.9 ± 0.2 ^b^	1.1 ± 0.0 ^a^
22:6 n-3	32.9 ± 1.0 ^c^	4.0 ± 0.2 ^a^	27.6 ± 2.4 ^b^	6.3 ± 0.2 ^a^
**PUFA**	**53.9 ± 1.5 ^d^**	**16.8 ± 1.0 ^a^**	**47.0 ± 2.3 ^c^**	**21.4 ± 0.7 ^b^**
**n-3 PUFA**	**48.0 ± 1.4 ^d^**	**13.1 ± 0.9 ^a^**	**41.9 ± 2.9 ^c^**	**17.7 ± 0.6 ^b^**
**n-6 PUFA**	**5.7 ± 0.2 ^c^**	**3 ± 0.1 ^a^**	**4.7 ± 0.6 ^b^**	**2.9 ± 0.1 ^a^**

Values are presented as the average ± standard deviation. In the same line, different letters represent significantly different arithmetic means (*p* < 0.05). PL, phospholipids; TAG, triacylglycerols.

**Table 5:** Total lipid classes profile of fish oil rich in DHA-PL before and after hydrolysis (expressed in % of total lipid content).

	Before hydrolysis (PreH_extract)	After hydrolysis (PostH_extract)
PL (%)	50.79 ± 4.92 ^b^	21.16 ± 2.74 ^a^
NL (%)	49.21 ± 4.92 ^a^	78.84 ± 2.74 ^b^
MAG	ND	ND
1,2 DAG	ND	ND
1,3 DAG + CH (%)	6.96 ± 1.01	6.03 ± 0.79
FFA (%)	11.50 ± 0.29 ^a^	46.04 ± 2.39 ^b^
TAG (%)	30.76 ± 4.13	26.76 ± 1.23

Values are presented as the average ± standard deviation. In the same line, different letters represent significantly different arithmetic means (*p* < 0.05). ND, not detected. PL, phospholipids; NL, non-polar lipids; MAG, monoacylglycerols; DAG, diacylglycerols; CH, cholesterol; FFA, free fatty acids; TAG, triacylglycerols.

**Table 6:** Phospholipid classes profile of fish oil rich in DHA-PL before and after hydrolysis (expressed in % of total phospholipids content).

	Before hydrolysis (PreH_extract)	After hydrolysis (PostH_extract)
LPC (%)	ND	25.19 ± 3.38
LPE (%)	ND	14.90 ± 3.70
PC (%)	57.76 ± 1.85 ^b^	21.69 ± 4.09 ^a^
PE (%)	42.24 ± 1.85	38.23 ± 4.52

Values are presented as the average ± standard deviation. In the same line, different letters represent significantly different arithmetic means (*p* < 0.05). ND, not detected.PL, phospholipids; NL, non-polar lipids; MAG, monoacylglycerols; DAG, diacylglycerols; CH, cholesterol; FFA, free fatty acids; TAG, triacylglycerols.

**Table 7:** Fatty acid profile in PL fractions present in extracts before and after enzymatic hydrolysis (expressed in % of total fatty acid).

	Before hydrolysis (PreH_extract)				After hydrolysis (PostH_extract)			
	PC	PE	LPC	LPE	PC	PE	LPC	LPE
14:0	1.4 ± 0.3 ^b^	0.3 ± 0.3 ^a^	-	-	0.2 ± 0.1 ^a^	0.1 ± 0.1 ^a^	0.2 ± 0.1 ^a^	0.4 ± 0.1 ^a^
16:0	19.5 ± 2.5 ^c^	9.4 ± 1.2 ^b^	-	-	1.3 ± 0.1 ^a^	1.2 ± 0.0 ^a^	0.6 ± 0.1 ^a^	1.0 ± 0.2 ^a^
18:0	5.8 ± 1 ^d^	7.9 ± 0.3 ^e^	-	-	1.1 ± 0.0 ^a,b^	1.4 ± 0.1 ^b,c^	0.5 ± 0.2 ^a,b^	2.5 ± 0.8 ^c^
**SFA**	**28.3 ± 2.7 ^d^**	**19.7 ± 1.9 ^c^**	**-**	**-**	**8.5 ± 0.8 ^b^**	**9.1 ± 0.9 ^b^**	**1.6 ± 0.4 ^a^**	**6.1 ± 0.6 ^b^**
16:1 n-7	0.9 ± 0.1 ^d^	0.6 ± 0.0 ^c^	-	-	0.2 ± 0.0 ^b^	0.3 ± 0.0 ^b^	0.1 ± 0.1 ^a^	0.2 ± 0.0 ^b^
18:1 n-9	6.1 ± 0.4 ^e^	4.0 ± 0.2 ^d^	-	-	3.2 ± 0.1 ^c,d^	3.1 ± 0.3 ^c^	0.7 ± 0.1 ^a,b^	1.1 ± 0.6 ^b^
18:1 n-7	1.6 ± 0.2 ^b^	3.5 ± 0.2 ^c^	-	-	1.0 ± 0.1 ^a^	1.0 ± 0.0 ^a^	1.2 ± 0.1 ^a,b^	0.6 ± 0.4 ^a^
20:1 n-9	2.4 ± 0.1 ^c^	3.9 ± 0.2 ^d^	-	-	0.6 ± 0.1 ^b^	0.2 ± 0.2 ^a,b^	0.6 ± 0.1 ^b,c^	0.4 ± 0.3 ^a,b^
22:1 n-11	0.2 ± 0.0 ^a,b^	0.1 ± 0.0 ^a,b^	-	-	0.1 ± 0.1 ^a^	0.2 ± 0.2 ^a,b^	0.1 ± 0.1 ^a,b^	0.4 ± 0.2 ^b^
22:1 n-9	0.0 ± 0.0	ND	-	-	ND	ND	ND	0.1 ± 0.1
24:1 n-9	0.0 ± 0.0	ND	-	-	ND	ND	ND	0.0 ± 0.0
**MUFA**	**12.0 ± 0.6 ^c^**	**12.7 ± 0.3 ^c^**	**-**	**-**	**5.6 ± 0.0 ^b^**	**5.9 ± 0.2 ^b^**	**2.8 ± 0.6 ^a^**	**3.1 ± 1.1 ^a^**
18:2 n-6	2.0 ± 0.2 ^c^	2.5 ± 0.1 ^d^	-	-	0.4 ± 0.0 ^b^	0.2 ± 0.2 ^a,b^	0.2 ± 0.2 ^a,b^	0.2 ± 0.2 ^a,b^
18:3 n-3	0.7 ± 0.0 ^c^	1.1 ± 0.0 ^d^	-	-	0.2 ± 0.0 ^b^	0.1 ± 0.1 ^a,b^	0.1 ± 0.1 ^a^	0.0 ± 0.1 ^a^
18:4 n-3	0.7 ± 0.0 ^a^	0.4 ± 0.0 ^a^	-	-	0.3 ± 0.0 ^a^	0.6 ± 0.4 ^a^	1.5 ± 0.1 ^b^	0.7 ± 0.6 ^a^
20:4 n-6	1.9 ± 0.2 ^d^	1.7 ± 0.1 ^c,d^	-	-	1.7 ± 0.1 ^c,d^	1.2 ± 0.1 ^a,b^	1.5 ± 0.1 ^b,c^	0.9 ± 0.2 ^a^
20:5 n-3	12.0 ± 0.7 ^d^	9.2 ± 0.4 ^c^	-	-	9.4 ± 0.3 ^c^	6.8 ± 0.6 ^b^	10.9 ± 0.2 ^c,d^	3.6 ± 1.6 ^a^
22:5 n-3	2.0 ± 0.2 ^c^	2.1 ± 0.0 ^c^	-	-	2.3 ± 0.0 ^c^	2.3 ± 0.2 ^c^	1.4 ± 0.2 ^b^	0.8 ± 0.4 ^a^
22:6 n-3	33.6 ± 2.6 ^a^	41.4 ± 1.5 ^b^	-	-	55.6 ± 1.4 ^c^	49.9 ± 0.8 ^c^	73.6 ± 1.8 ^d^	73.4 ± 4.5 ^d^
**PUFA**	**56.3 ± 3.2 ^a^**	**63.8 ± 1.4 ^b^**	**-**	**-**	**78.3 ± 1.2 ^d^**	**73.1 ± 0.6 ^c^**	**94.1 ± 0.7 ^f^**	**86.6 ± 0.6 ^e^**
**n-3 PUFA**	**50.4 ± 3.2 ^a^**	**57.1 ± 1.3 ^b^**	**-**	**-**	**74.1 ± 1.0 ^c^**	**69.6 ± 1.1 ^c^**	**89.1 ± 1.1 ^e^**	**80.3 ± 3.2 ^d^**
**n-6 PUFA**	**5.4 ± 0.1 ^b^**	**6.2 ± 0.2 ^b^**	**-**	**-**	**3.5 ± 0.4 ^a,b^**	**2.7 ± 0.3 ^a,b^**	**4.9 ± 0.2 ^b^**	**6.2 ± 3.8 ^b^**
**n-3/n-6**	**9.3 ± 0.6 ^a^**	**9.2 ± 0.1 ^a^**	**-**	**-**	**21.2 ± 1.8 ^b,c^**	**26.4 ± 3.6 ^c^**	**18.4 ± 1.1 ^b,c^**	**16.1 ± 7.9 ^a,b^**

Values are presented as the average ± standard deviation. In the same line, different letters represent significantly different arithmetic means (*p* < 0.05). ND, not detected. PC, phosphatidylcholine; PE, phosphatidylethanolamine; LPC, lysophosphatidylcholine; LPE, lysophosphatidylethanolamine.

## Data Availability

The datasets generated for this study are available on request to the corresponding author.
